# Olmesartan medoxomil self-microemulsifying drug delivery system reverses apoptosis and improves cell adhesion in trinitrobenzene sulfonic acid-induced colitis in rats

**DOI:** 10.1080/10717544.2022.2086939

**Published:** 2022-06-29

**Authors:** Hussam Murad, Osama Ahmed, Thamer Alqurashi, Mostafa Hussien

**Affiliations:** a Department of Pharmacology, Faculty of Medicine, Rabigh campus, King Abdulaziz University, Jeddah, Saudi Arabia; b Department of Pharmaceutics, Faculty of Pharmacy, King Abdulaziz University, Jeddah, Saudi Arabia; c Department of Chemistry, Faculty of Science, King Abdulaziz University, Jeddah, Saudi Arabia

**Keywords:** Crohn's disease, olmesartan medoxomil, drug delivery, colitis, E-cadherin, Caspase, Bcl-2, MMP-9

## Abstract

Olmesartan medoxomil (OM) is an angiotensin receptor blocker. This study aimed to investigate the effects of OM self-microemulsifying drug delivery system (OMS) in trinitrobenzene sulfonic acid (TNBS)-induced acute colitis in rats. Besides two control groups, five TNBS-colitic-treated groups (*n* = 8) were given orally sulfasalazine (100 mg/kg/day), low and high doses of OM (3.0 and 10.0 mg/kg/day) (OML and OMH) and of OMS (OMSL and OMSH) for seven days. A colitis activity score was calculated. The colon was examined macroscopically. Colonic levels of myeloperoxidase, tumor necrosis factor-α (TNF-α), interleukin-6 (IL-6), malondialdehyde, and reduced glutathione were measured. Plasma and colonic olmesartan levels were measured. Colonic sections were subjected to hematoxylin and eosin staining and immunohistochemical staining for E-cadherin, caspase-3, and matrix metalloproteinase-9 (MMP-9). Protein expression of E-cadherin, Bcl-2 associated X protein (Bax), and B-cell lymphoma 2 (Bcl-2), and cleaved caspase-3 by Western blot was done. TNBS-colitic rats showed increased colonic myeloperoxidase, TNF-α, IL-6, and malondialdehyde, decreased colonic glutathione, histopathological, immunohistochemical, and protein expression alterations. OMS, compared with OM, dose-dependently achieved higher colonic free olmesartan concentration, showed better anti-inflammatory, antioxidant, and anti-apoptotic effects, improved intestinal barrier, and decreased mucolytic activity. OMS more effectively up-regulated the reduced Bcl-2, Bcl-2/Bax ratio, and E-cadherin expression, and down-regulated the overexpressed Bax, cleaved caspase-3, and MMP-9. OMSL exerted effects comparable to OMH. Sulfasalazine exerted maximal colonic protective effects and almost completely reversed colonic damage, and OMSH showed nearly similar effects with non-significant differences in-between or compared with the normal control group. In conclusion, OMS could be a potential additive treatment for Crohn's disease colitis.

## 1. Introduction

Several factors are involved in the pathogenesis of inflammatory bowel disease (IBD). Generally, Crohn’s disease has a T helper 1 (Th1) cytokine profile where the Th1 cells produce pro-inflammatory cytokines such as interferon-γ (IFN-γ), interleukin 2 (IL-2) and tumor necrosis factor α (TNF-α) (Brand, 2009). Recently, the epithelial-mesenchymal transition (EMT)-related proteins have been found important in the progress of intestinal fibrosis. E-cadherin is an EMT-related protein that is considered a critical cell adhesion molecule. The reduction of its expression in the surface epithelium may remodel the adherens junctions between epithelial cells (Li et al., 2002). E-cadherin acts as a transmembrane junctional adhesion molecule in the intestinal epithelial cells (Sun et al., 2015). Moreover, up-regulated expression of matrix metalloproteinase-9 (MMP-9) has been observed in experimental colitis (Garg et al., 2009) and in human inflammatory bowel disease (IBD) where it induces mucosal proteolysis and epithelial ulcerations (Naito & Yoshikawa, 2002). Also, the increased apoptosis of intestinal epithelial cells impairs the epithelial barrier mechanism and contributes to injury (Becker et al., 2013). Activation of apoptosis is indicated by upregulation of caspase-3 which is the major executioner of caspase (Arab et al., 2014). The current therapies for IBD have several side effects and variable efficacy besides the excessive cost of the biological medications. Thus, finding effective, safe, and inexpensive medications for IBD is critical (Yousefi-Ahmadipour et al., 2020). De novo drug discovery is costly and time-consuming, thus use of drug repurposing of already-marketed drugs as an alternative approach is a preferred strategy. It allows rapid, relatively inexpensive, and safe drug discovery (Mehndiratta et al., 2016).

The renin angiotensin aldosterone system (RAS) is a key controller of blood volume, peripheral resistance, and blood pressure. It acts in a prolonged manner to increase blood pressure in response to reduced renal blood pressure, decreased sodium delivery to the distal convoluted tubule, and/or activation of adrenergic β receptors. The responsible mechanisms include increasing sodium and reabsorption and vasoconstriction. Activation of the RAAS leads to the conversion of angiotensin I to angiotensin II by the angiotensin converting enzyme (ACE) resulting in elevation of blood pressure, cellular proliferation, inflammation, and fibrosis which may result in many acute and chronic diseases (Patel et al., 2017). Angiotensin II acts mainly through binding to angiotensin II type I (AT1) G protein-coupled receptors leading to arteriolar vasoconstriction, increased total peripheral resistance, and increased blood pressure. In the renal proximal convoluted tubule, angiotensin II increases sodium reabsorption increasing the arterial pressure. Angiotensin II acts on the adrenal cortex stimulating the release of aldosterone which binds to its nuclear receptors to alter gene transcription. It causes sodium retention and potassium loss at the renal distal convoluted tubule and collecting duct by stimulating the insertion of luminal sodium channels and basolateral Na^+^/K^+^ ATPase proteins. Also, angiotensin II stimulates thirst and release of the antidiuretic hormone and diminishes sensitivity of the baroreceptor reflex. All these effects lead to increased vascular tone and total body sodium and water (Fountain & Lappin, 2017). Unfortunately, the RAS can be activated inappropriately in several conditions e.g. renal artery stenosis. ACE inhibitors (ACEIs), angiotensin II receptor blockers (ARBs), and aldosterone antagonists are clinically used to antagonize the effects of the RAS. Thus, ACEIs and ARBs cause vasodilatation, inhibition of aldosterone secretion, prevention of myocardial remodeling, and anti-adrenergic effects and hence they are indicated for the treatment of chronic heart failure, hypertension, especially with diabetes mellitus, acute myocardial infarction, and diabetic nephropathy (Nehme et al., 2019). Angiotensin II (Ang II) is a key pro-inflammatory, pro-oxidative, and pro-apoptotic factor. The AT1 receptor (AT1R) is expressed in several immune and blood cells and the RAS is involved in the pathogenesis of IBD (Wang et al., 1999; Fändriks, 2010). Thus, inhibition of angiotensin II could attenuate inflammation, apoptosis, and oxidant stress and subsequently improves acute colitis. The ARBs are anti-hypertensive medications with anti-inflammatory and anti-fibrotic properties which are mediated *via* antagonizing effects of the classical (ACE/Ang II/AT1R) and upregulating the alternative tissue-protective ACE2/Ang-(1–7)/Mas receptor pathway with elevated tissue Ang (1–7) (Ferrario et al., 2005). Recently, another cardiovascular protective axis of the RAS was discovered, established by the alatensins which are angiotensin peptides having alanine instead of aspartate in their amino terminal. The Ala-Ang-(1–7) or alamandine was found to act as an endogenous ligand of the Mas-related G protein-coupled receptor (MrgD) exerting many neuronal effects and alamandine-induced vasodilation (Santos et al., 2019; Schleifenbaum, 2019; ).

Olmesartan medoxomil (OM) is a selective AT1 receptor blocker with almost no interference with the AT2 and AT4 receptors. It has an inverse agonist activity, upregulates the ACE2/Ang-(1–7)/Mass receptor pathway, inhibits ACE, and reduces plasma concentration of angiotensin II. But unfortunately, it has low bioavailability due to its highly lipophilic nature and efflux by gut drug resistance pumps (Kobayashi et al., 2000). This low bioavailability was enhanced by a self-micro emulsifying drug delivery system (SMEDDS) which is an OM lipophilic formulation rapidly absorbed from the intestine and hence does not cause sprue-like enteropathy; which could occur as a side effect of the regular OM (Komesli et al., 2019). OM was reported to have protective, anti-inflammatory, and antioxidant effects in dextran sodium sulfate (DSS)-induced colitis and acetic acid-induced colitis in rats (Nagib et al., 2013; Saber et al., 2019). There are numerous clinical studies showing that treatment with OM may induce severe sprue-like enteropathy and collagenous colitis, but interestingly, discontinuation of OM led to dramatic improvement of the symptoms. Severe olmesartan-induced sprue-like enteropathy and colitis were diagnosed in a 63-year-old female who presented with refractory diarrhea and weight loss (Bashari, 2020). More recently, a case of olmesartan-induced lymphocytic colitis was diagnosed in an 80-year-old female hypertensive patient with a history of myocardial infarction who presented with severe diarrhea (Zimmer & Heinrich, 2021). Moreover, a case of sprue-like enteropathy and collagenous colitis was observed in a 73-year-old man with severe diarrhea and weight loss over the last two months (Kaneko et al., 2021). Olmesartan-induced enteropathy was also detected in a 76-year-old female complaining of chronic diarrhea and significant weight loss (Sotiropoulos et al., 2021).

Adequate blood flow is a key element in the protection and regeneration of the gastrointestinal organs. The drop in gastric mucosa blood flow increases its sensitivity to noxious factors and promotes the development of a gastric ulcer. The superficial mucosal injury is followed by increased blood flow to support the healing process and prevent the development of deep lesions *via* increasing the supply of oxygen and bicarbonate and removing H^+^ and aggressive agents. Mucosal ischemia contributes to gastric ulceration and vessels are damaged in gastric ulcers during healing the blood flow returns to normal and thus healing is affected by stimulation or inhibition of angiogenesis in the granulation tissue (Sørbye & Svanes, 1994). A lot of studies proved that improvement of the gastric blood flow exhibits gastroprotective and healing promoting effects. Surprisingly, histamine was reported to protect against stress-induced gastric damage by increasing gastric blood flow and inhibiting the activation of the cytokine cascade (Warzecha et al., 2001). Treatment with ghrelin enhanced the healing of acetic acid-induced gastric and duodenal ulcers in rats by increasing mucosal cell proliferation and blood flow (Ceranowicz et al., 2009). Similar protective and healing-promoting effects of sufficient organ blood flow have been also found in the oral cavity in the healing of gingival ulcers by exogenous ghrelin (Cieszkowski et al., 2017), and in the duodenum where ghrelin increased the healing rate of duodenal ulcers (Warzecha et al., 2012). Moreover, pancreatic ischemia followed by reperfusion caused acute necrotizing pancreatitis followed by regeneration within four weeks (Dembiński et al., 2001). The extract of grapefruit seed protected against acute pancreatitis induced by ischemia/reperfusion *via* improving the pancreatic blood flow (Dembinski et al., 2004). In addition, induction of colitis was associated with a reduced blood flow, while measures protecting mucosa and speeding up recovery were associated with its improvement. These effects were observed in different models of colitis where ghrelin reversed the colitis-induced decrease in blood flow in DSS-induced colitis (Matuszyk et al., 2015) and in acetic acid-induced colitis (Maduzia et al., 2015). Also, the synergistic therapeutic effect of rifaximin and mutaflor in acetic acid-induced colitis was associated with a substantial reversal of the acetic acid-induced decrease in mucosal blood flow (Dembiński et al., 2016). In trinitrobenzene sulfonic acid (TNBS)-induced colitis, treatment with obestatin (a ghrelin gene-encoded peptide) reduced severity of colitis and was associated with increased colonic mucosal blood flow (Konarska et al., 2018).

Taken together, the current study was designed to investigate the effects of an OM self-microemulsifying drug delivery system (OMS), compared with OM, in TNBS-induced acute colitis in rats, a model which resembles Crohn's disease like colitis (Randhawa et al., 2014). We hypothesize that the OM nanoformulation could preserve the epithelial integrity in TNBS-induced acute colitis in rats more effectively than the regular OM.

## 2. Materials and methods

### 2.1. Formulation of OMS nanoformula

OM was prepared in a SNEDDS formula composed of oil part (oleic acid 10% w/w), surfactant mixture (tween 80 and span 85, 1:1 weight ratio 50% w/w), and cosurfactant (labrasol 40% w/w). The OMS formula was prepared as early as reported (Murad et al., 2020). The concentration of OM in SNEDDS components was 10 mg/g formula.

### 2.2. Characterization of OMS nanoformula

#### 2.2.1. Globule size and zeta potential evaluation

The OMS globule size and zeta potential were evaluated utilizing Zetasizer Nano ZSP (Malvern Panalytical Ltd, Malvern, UK) after dilution with deionized water.

#### 2.2.2. Transmission electron microscope (TEM) examination of OMS nano-formula

OMS formula was investigated using JEOL GEM-1010 (JEOL Ltd., Akishima, Tokyo, Japan) TEM at 80 kV in the Regional Center for Mycology and Biotechnology, Cairo, Egypt. One drop of OMS formula was allowed to be dried after spreading on the TEM grid at room temperature, then stained using 1% phosphotungstic acid and dried before visualization.

### 2.3. Animals

The study was ethically approved by the Ethics Committee at the Faculty of Pharmacy, King Abdulaziz University, Reference No “PH-1442-76.” All animal experiments complied with the guidelines of the National Institutes of Health guide for the care and use of laboratory animals. Sprague-Dawley male rats (200–250 g), acquired from the animal house, Faculty of Pharmacy, were housed in cages at 22 °C, 55 ± 5% humidity, in a 12 hours' light-dark cycle for a 7-day acclimation period before starting the experiment with unrestricted access to standard food and clean water.

### 2.4. Induction of acute TNBS-colitis and experimental design

The rats were food-deprived for a day, and colitis was induced as previously described (Wang et al., 2010). Rats were anesthetized with ether and then a 5% TNBS (Sigma, St Louis, USA) (100 mg/kg) dissolved in 0.25 mL of 50% ethanol (v/v) (2:1) was administered by a polyethylene catheter introduced rectally to 6–8 cm from the anus. Rats were set in a head-down position for 30 seconds to avoid leakage of the solution (Lu et al., 2017). Rats were randomly divided into seven groups (*n* = 8) including the normal control (NC) group, TNBS-colitic (positive control, PC) group, and five TNBS-colitic-treated groups which were orally given sulfasalazine (SSZ, 100 mg/kg/day, a reference treatment for IBD) (Yousefi-Ahmadipour et al., 2020), low and high doses of OM (OML and OMH: 3.0 and 10.0 mg/kg/day (Nagib et al., 2013)) as a raw drug and as an OMS (OMSL and OMSH). OM was suspended in distilled water containing 0.25% carboxymethyl cellulose (Gorain et al., 2014; Komesli et al., 2019). The treatments were given for 7 days starting 4 hours after TNBS administration (Lu et al., 2017). Rats were evaluated for the colitis activity score. At end of the treatment period, blood was collected, and rats were sacrificed. The colon was excised, cleaned with ice-cold saline, measured for weight and length, and the macroscopic appearance was evaluated. Parts of the colon were homogenized and activities/levels of myeloperoxidase (MPO), tumor necrosis factor-α (TNF-α), interleukin-6 (IL-6), malondialdehyde (MDA), and reduced glutathione (GSH) were measured. The concentrations of olmesartan in plasma and colonic homogenate were measured by high performance liquid chromatography/mass spectrometry (HPLC/MS). Samples of the colon were obtained for hematoxylin and eosin (H&E) and immunohistochemical staining (Joo et al., 2015)). All evaluations were made by blinded observers.

### 2.5. Evaluation of the disease activity score

The colitis activity index was measured to evaluate the activity of intestinal inflammation. The scores for weight loss, stool consistency, and fecal blood were calculated to give a disease activity score (DAS) as previously described (Wang et al., 2014). The combined score was calculated according to the following parameters: (1) No weight loss was scored 0 while 1–15% was scored 1, and more than 15% was scored 2, (2) No fecal blood was equal to 0 while less than or equal to half of the stool surface was equal to 1, and more than 50% was equal to 2, and (3) Normal fecal consistency was equal to 0, while 1 was given to loose stools, and 2 expressed diarrhea.

### 2.6. Colonic macroscopic examination

The colon was measured for weight and length. The degree of colonic damage regarding edema, ulceration, bleeding, and necrosis was expressed as mild, moderate, or severe (Ghia et al., 2006).

### 2.7. Colonic measurements

The colonic samples were homogenized using phosphate-buffered saline and a TissueLyser II (Qiagen) at 4 °C to yield a 10% homogenate. Following centrifugation at 4 °C and 10,000 rpm for 15 min., the supernatant was kept at −80 °C till analysis. The colonic protein content was determined by using the Bradford method (Bradford, 1976). The activities and/or levels of MPO (a marker for neutrophil infiltration), TNF-α, IL-6, MDA, and GSH were measured using ELISA kits (MyBioSource, Inc. CA, USA) according to the manufacturer's guidelines and were expressed/mg protein.

### 2.8. Histopathological examination

The colonic parts were fixed in 10% buffered formalin and embedded in paraffin. Sections (3–5 µm) were prepared and stained with H&E. The degree of inflammation was expressed as mild, moderate, or severe (Wirtz et al., 2007).

### 2.9. Immunohistochemical examination for E-cadherin, caspase-3, and matrix metalloproteinase-9 (MMP-9)

Colonic sections were immunohistochemically stained for E-cadherin and Caspase-3 (ab13847; Abcam; 1:500 dilution). Briefly, 3–5 µm paraffin sections were deparaffinized, rehydrated, and submerged in hydrogen peroxide. After incubation with the primary antibodies at 4 °C for 12 hours, secondary antibodies IgG (ab6721; Abcam; 1:1000 dilution) were applied to slides for an hour at room temperature. The sections were colored with a diaminobenzidine kit followed by counterstaining with hematoxylin and were examined by a light microscope (Sun et al., 2015; t al., 2020). For MMP-9 immunostaining, the sections were probed with rabbit and mouse monoclonal anti-MMP-9 (1:150 dilution), detected with sheep anti-rabbit or anti-mouse IgG fluorescein isothiocyanate (1:500 dilution), and examined under a fluorescent microscope (Baugh et al., 1999).

### 2.10. Protein expression of E-cadherin, Bcl-2 associated X protein (Bax), B-cell lymphoma 2 (Bcl-2), and cleaved caspase-3 by western blot

The colonic tissue was homogenized by incubation for 30 minutes in ice-cold radioimmunoprecipitation assay buffer with protease and phosphatase inhibitor cocktails, followed by centrifugation for 20 min at 4 °C (4000 rpm). The supernatant was used for the assay of protein content by the BCA protein assay kit (Cat. No. 355526, MyBioSource, Inc. CA, USA). The protein lysates (80 *µ*g/lane) were separated on a precast protein gel (4–20%) of sodium dodecyl sulfate-polyacrylamide gel electrophoresis (SDS-PAGE). After electrophoresis for 1 h, the separated proteins were transferred onto the activated polyvinylidene difluoride membrane (Cat # ab133411, Abcam, Cambridge, UK). The whole membrane was blocked by 5% nonfat dry milk at room temperature for 1 h. The membrane was then cut into four pieces at molecular weights of 80, 35 and 20 kDa. The membrane part (< 20 kDa) was incubated overnight with the primary rabbit polyclonal anti-caspase 3 (cleaved form) at 1:500 (Cat # AB3623, Sigma Aldrich Comp., Missouri, USA). The piece (20–35 kDa) was incubated with the primary mouse monoclonal anti-Bax at 1:500 (Cat # sc-7480, Santa Cruz Biotechnology, Inc., Texas, USA). The membrane (35 − 80 kDa) was probed with rabbit anti-β-actin antibody (Cat # ab8227, Abcam, Cambridge, UK), as a loading control. The piece (> 80 kDa) was incubated with the primary mouse monoclonal anti-E cadherin at 1:500 (Cat # sc-21791, Santa Cruz Biotechnology, Inc., Texas, USA). After washing, the membranes (probed against cleaved caspase3 & β-actin) were incubated with goat anti-rabbit HRP-conjugated secondary antibody at 1:5000 (Cat # ab6721, Abcam, Cambridge, UK) for 1 hour at room temperature. However, the other two membrane pieces (probed against E-cadherin and Bax) were incubated with anti-mouse HRP-conjugated secondary antibody at 1:5000 (Cat # sc-525409, Santa Cruz Biotechnology, Inc., Texas, USA). The membranes were then developed by an enhanced chemiluminescence (ECL) kit (GE Healthcare, Piscataway, NJ, USA) and the protein bands were visualized on X-ray film. Afterwards, the membrane piece (20–35 kDa) was stripped, followed by re-probing with primary mouse monoclonal anti-Bcl-2 at 1:500 (Cat # sc-7382, Santa Cruz Biotechnology, Inc., Texas, USA), and the same steps were repeated until developing the membrane (Burnette, 1981; Sambrook, 1989)

### 2.11. Determination of olmesartan concentration in plasma and colonic homogenate

The concentrations of the olmesartan in plasma and the colonic homogenate were measured by HPLC/MS (Sharma & Pancholi, 2010). The sample extraction and analysis were done as previously reported with slight modifications (Liu et al., 2010; Murad et al., 2020).

### 2.12. Statistical analysis

Data were expressed as means ± SEM and analyzed using SPSS version 22. One-way ANOVA and Tukey's post hoc tests were used to evaluate differences among groups. *P* < .05 was considered statistically significant.

## 3. Results

### 3.1. Formulation and characterization of OMS nanoformula

**Figure 1.**
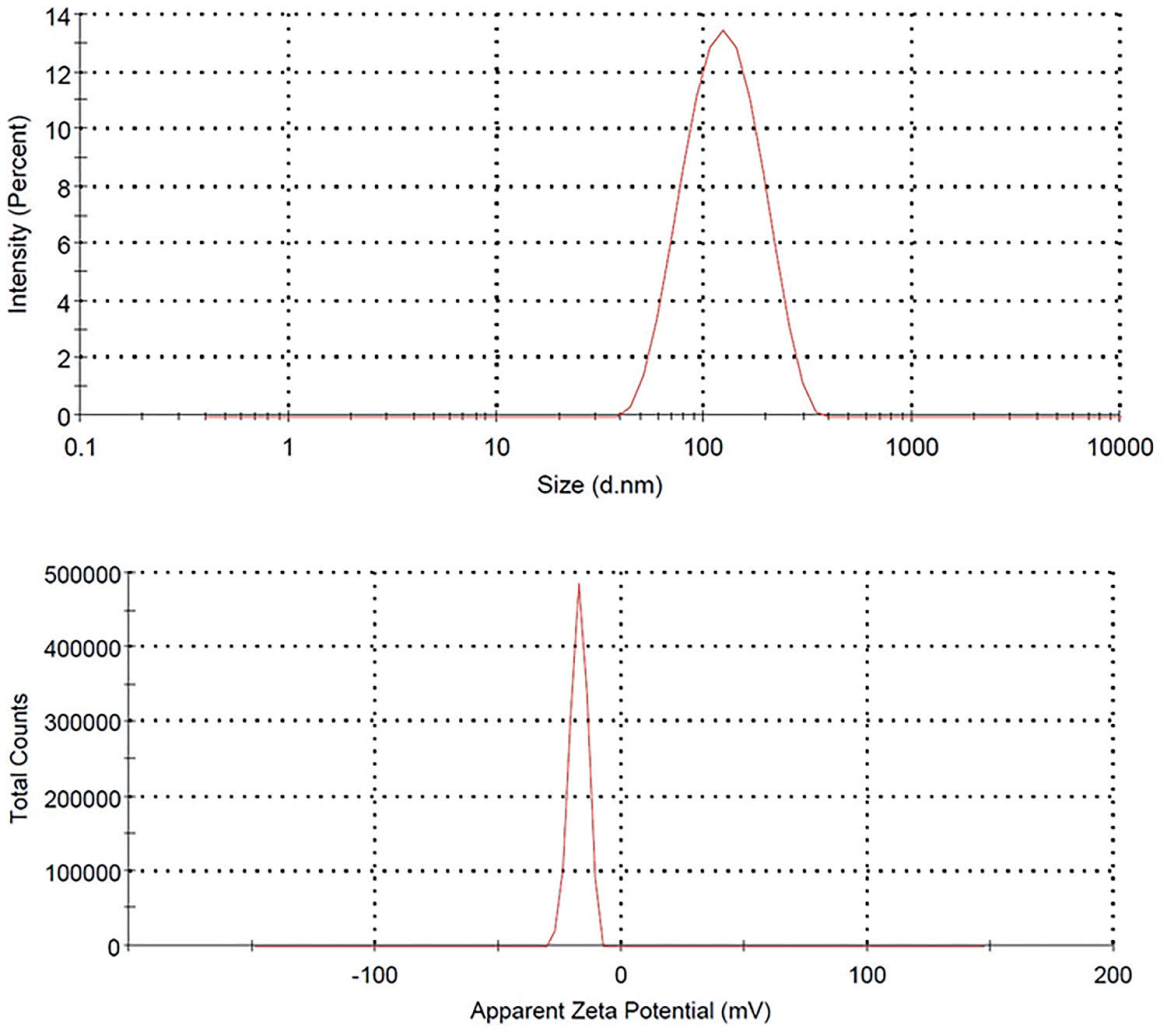
Globule size and apparent zeta potential data of the olmesartan medoxomil self-microemulsifying drug delivery system (OMS) nanoformula.

**Figure 2.**
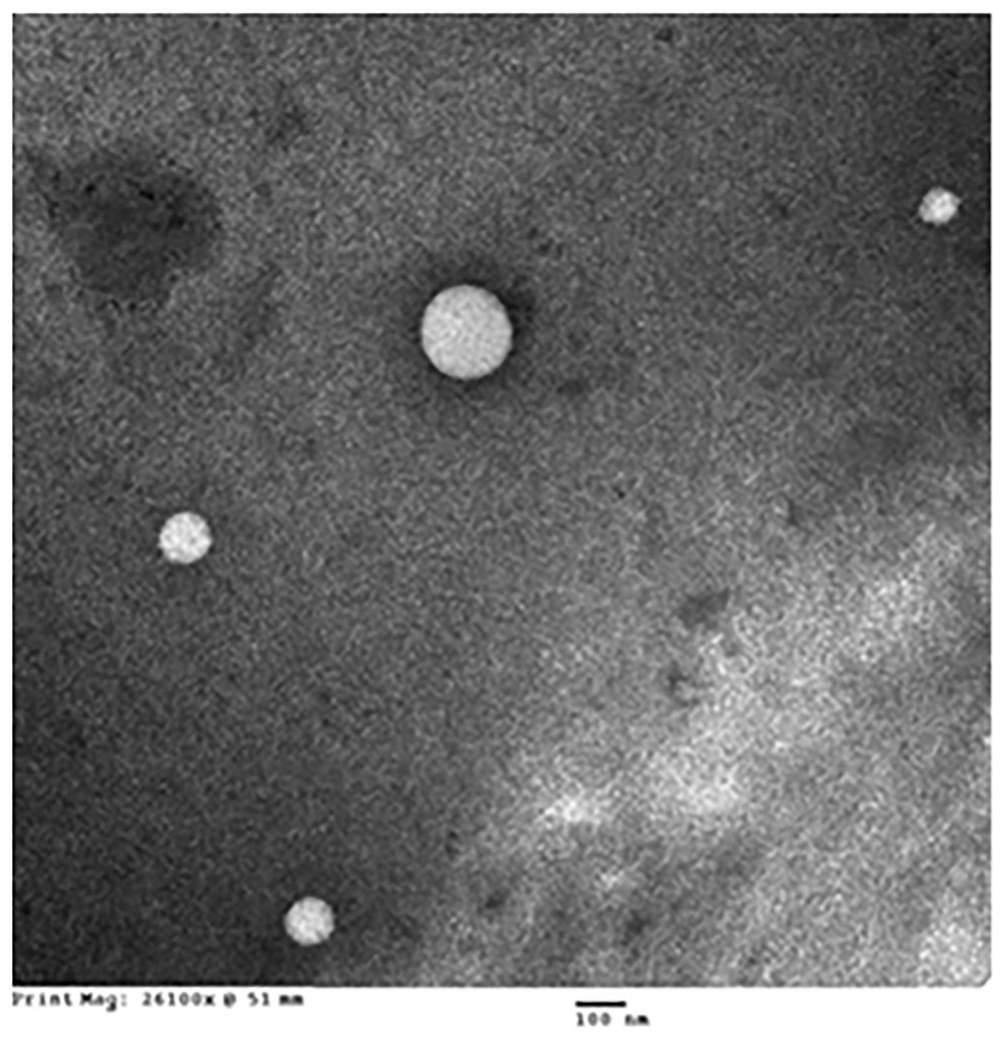
Transmission electron microscope (TEM) images of olmesartan medoxomil self-microemulsifying drug delivery system (OMS) nanoformula.

The prepared formula demonstrated a nearly clear solution on mixing with water. The distribution of OMS within the aqueous dispersion medium as nano-sized globules is attributed to the almost clear dispersion formed after mixing the OMS formula with water. Size analysis of the formed OMS globules revealed globule sizes of 131.4 ± 12.1 nm and zeta potential values of −17.5 ± 3.6 mV (Figure 1). The TEM investigation of the OMS formula (Figure 2) revealed spherical structures with a relative globule comparable size measured by Zetasizer Nano ZSP (131.4 ± 12.1 nm) taking into consideration the drying process during the preparation of the OMS formula for the TEM investigation.

### 3.2. Disease activity score (DAS)

The TNBS-colitic rats showed an increased DAS compared with NC. All treatments significantly reduced this activity. The OML exerted mild improvement, while the OMH and OMSL displayed moderate improvement with non-significant differences in-between. The DAS was maximally decreased in the SSZ and OMSH treated groups with non-significant differences in-between or compared with the NC group (Table 1).

**Table 1.** Effects of olmesartan medoxomil and its nanoformula on the disease activity score, and colon weight/length ratio (mg/cm) in trinitrobenzene sulfonic acid-induced acute colitis in rats (*n* = 8).

	NC	PC	SSZ	OML	OMH	OMSL	OMSH
Disease activity score	0.28 ± 0.04	6.74 ± 0.31	0.45 ± 0.06 ^#, $^	5.69 ± 0.26 ^*, ^^	3.01 ± 0.25 ^*, #^	2.51 ± 0.16 ^*, #^	0.64 ± 0.07 ^#, $^
Colon weight/length ratio (mg/cm)	69.83 ± 3.39	204.98 ± 10.32	78.23 ± 2.11^#, $^	176.63 ± 3.49 ^*, ^^	121.50 ± 6.91 ^*, #^	114.30 ± 2.76 ^*, #^	81.31 ± 1.88 ^#, $, @^

Data are expressed as mean ± SEM. NC: Normal control, PC: Positive control, SSZ: Sulfasalazine, OML: Olmesartan medoxomil low dose, OMH: Olmesartan medoxomil high dose, OMSL: Olmesartan medoxomil self-microemulsifying drug delivery system low dose, OMSH: Olmesartan medoxomil self-microemulsifying drug delivery system high dose.

Disease activity score: *P < .001 vs. NC, ^#^*P* < .001 vs. PC, ^^^*P* < .01 vs. PC (*p* = .005), ^$^*P* < .001 vs. OML, OMH, OMSL.

Colon weight/length ratio: *P < .001 vs. NC, ^#^*P* < .001 vs. PC, ^^^*P* < .01 vs. PC (*p* = .007), ^$^*P* < .001 SSZ vs. OML, OMH, OMSL and OMHS vs. OML, OMH, ^@^*P* < .01 (*p* = .001) OMHS vs. OMSL.

### 3.3. Colonic macroscopic examination

**Figure 3.**
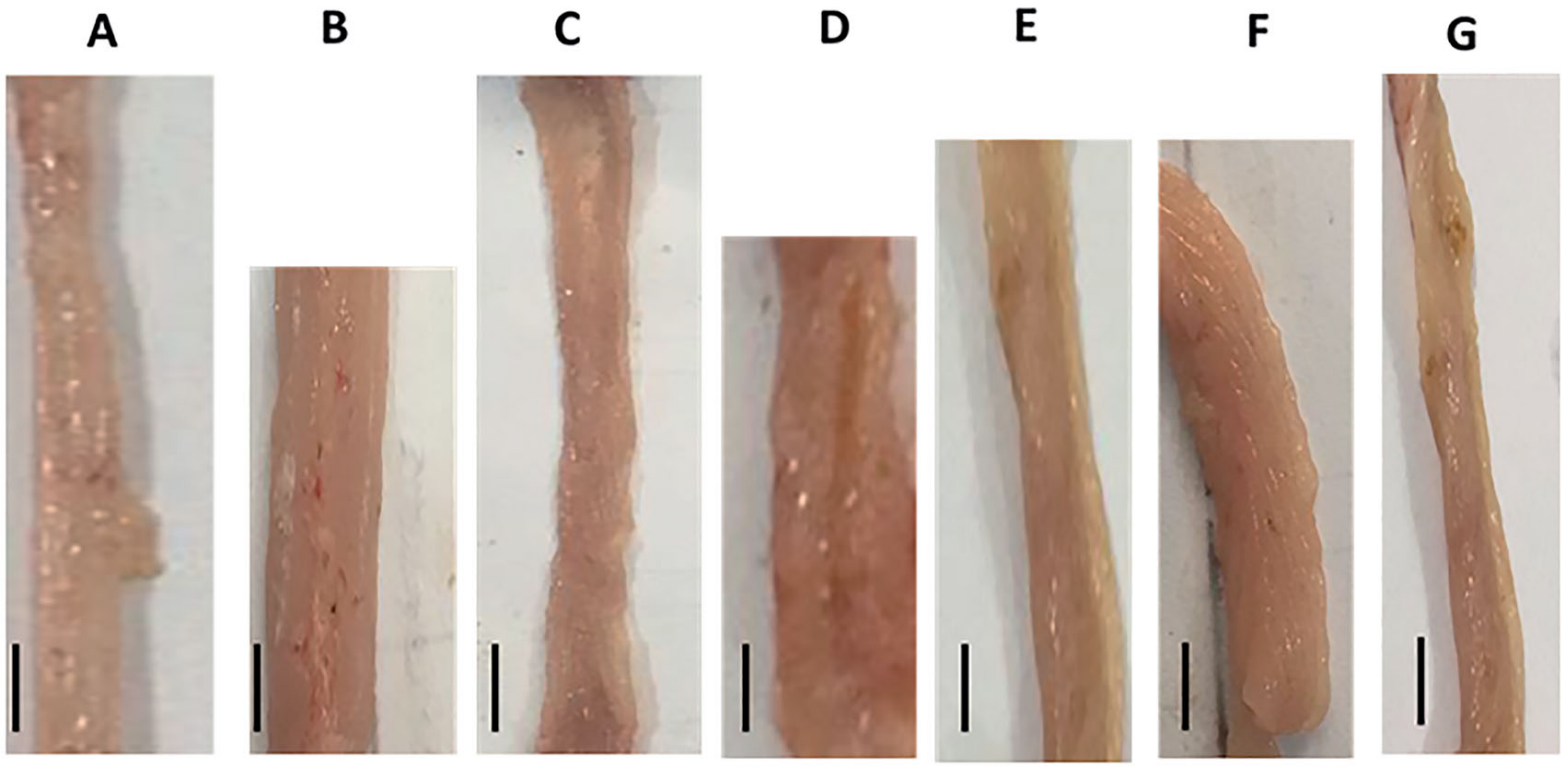
Olmesartan medoxomil self-microemulsifying drug delivery system (OMS) nanoformulation improves the colonic macroscopic damage in trinitrobenzene sulfonic acid-induced acute colitis in rats. The scale represents 1 cm. (A) Normal control, (B) Positive control, (C) Sulfasalazine, (D) Olmesartan medoxomil low dose, (E) Olmesartan medoxomil high dose, (F) Olmesartan medoxomil self-microemulsifying drug delivery system low dose, (G) Olmesartan medoxomil self-microemulsifying drug delivery system high dose.

The TNBS-colitic rats showed colonic damage manifested by inflammation, mucosal edema, bleeding, erosions, and necrosis (Figure 3) and elevated colon weight/length ratios. All treatments significantly improved these changes. The OML exerted mild improvement, while the OMH and OMSL displayed moderate improvement with non-significant differences in-between. The colonic macroscopic appearance was greatly improved in the SSZ and OMSH treated groups with non-significant differences in-between or compared with the NC group (Table 1).

### 3.4. Colonic measurements

The TNBS-colitic rats showed significant increases in the activities or contents of MPO, TNF-α, IL-6, and MDA and a significant reduction of GSH content in the colon homogenate. All treatments significantly improved these changes. The OML exerted mild improvement, while the OMH and OMSL displayed moderate improvement with non-significant differences in-between. The most significant decreases in the activities or contents of colonic MPO, TNF-α, IL-6, and MDA, and elevation of GSH content occurred in the SSZ and OMSH groups with non-significant differences in-between or compared with the NC group (Table 2).

**Table 2.** Effects of olmesartan medoxomil and its nanoformula on contents of myeloperoxidase (MPO), tumor necrosis factor-α (TNF-α), interleukin-6 (IL-6), malondialdehyde (MDA), and reduced glutathione (GSH) in colon homogenate in trinitrobenzene sulfonic acid-induced acute colitis rats (*n* = 8).

	NC	PC	SSZ	OML	OMH	OMSL	OMSH
MPO activity (U/mg protein)	6.18 ± 0.29	24.83 ± 1.19	8.01 ± 0.30 ^#, $, @^	20.90 ± 1.06 ^*, ^^	14.52 ± 0.61 ^*, #^	12.53 ± 0.43 ^*, #^	7.02 ± 0.29 ^#, $^
TNF-α (pg/mg protein)	16.26 ± 1.15	81.69 ± 2.35	20.49 ± 0.73 ^#, $^	69.98 ± 3.50 ^*, ^^	51.45 ± 2.47 ^*, #^	49.86 ± 0.75 ^*, #^	24.06 ± 1.21 ^#, $^
IL-6 (pg/mg protein)	56.47 ± 1.40	181.29 ± 3.20	65.06 ± 1.46 ^#, $^	160.57 ± 5.73 ^*, ^^	122.43 ± 4.29 ^*, #^	115.02 ± 3.23 ^*, #^	70.10 ± 3.73 ^#, $^
MDA (nmol/mg protein)	60.83 ± 2.14	244.19 ± 6.40	78.23 ± 2.11 ^#, $^	221.25 ± 6.23 ^*, ^^	149.57 ± 2.26 ^*, #^	133.78 ± 6.88 ^*, #^	76.16 ± 1.46 ^#, $^
GSH (nmol/mg protein)	43.09 ± 1.69	13.17 ± 0.64	39.75 ± 1.38 ^#, $^	19.77 ± 0.55 ^*, ^^	29.03 ± 1.44 ^*, #^	27.10 ± 0.95 ^*, #, @^	41.56 ± 2.14 ^#, $^

Data are expressed as mean ± SEM. NC: Normal control, PC: Positive control, SSZ: Sulfasalazine, OML: Olmesartan medoxomil low dose, OMH: Olmesartan medoxomil high dose, OMSL: Olmesartan medoxomil self-microemulsifying drug delivery system low dose, OMSH: Olmesartan medoxomil self-microemulsifying drug delivery system high dose.

For MPO: *P < .001 vs. NC, ^#^*P* < .001 vs. PC, ^^^*P* < .01 vs. PC (*p* = .004), ^$^*P* < .001 vs. OML, OMH, OMSL, ^@^*P* < .01 (*p* = .001) vs. OMSL.

For TNF-α: *P < .001 vs. NC, ^#^*P* < .001 vs. PC, ^^^*P* < .01 vs. PC (*p* = .002), ^$^*P* < .001 vs. OML, OMH, OMSL.

For IL-6: *P < .001 vs. NC, ^#^*P* < .001 vs. PC, ^^^: *P* < .01 vs. PC (*p* = .003), ^$^*P* < .001 vs. OML, OMH, OMSL.

For MDA: *P < .001 vs. NC, ^#^*P* < .001 vs. PC, ^^^: *P* < .05 vs. PC (*p* = .013), ^$^*P* < .001 vs. OML, OMH, OMSL.

For GSH: *P < .001 vs. NC, ^#^*P* < .001 vs. PC, ^^^*P* < .05 vs. PC (*p* = .020), ^$^*P* < .001 vs. OML, OMH, OMSL, ^@^*P* < .01 (*p* = .007) vs. OML.

### 3.5. Colonic histopathological examination

**Figure 4.**
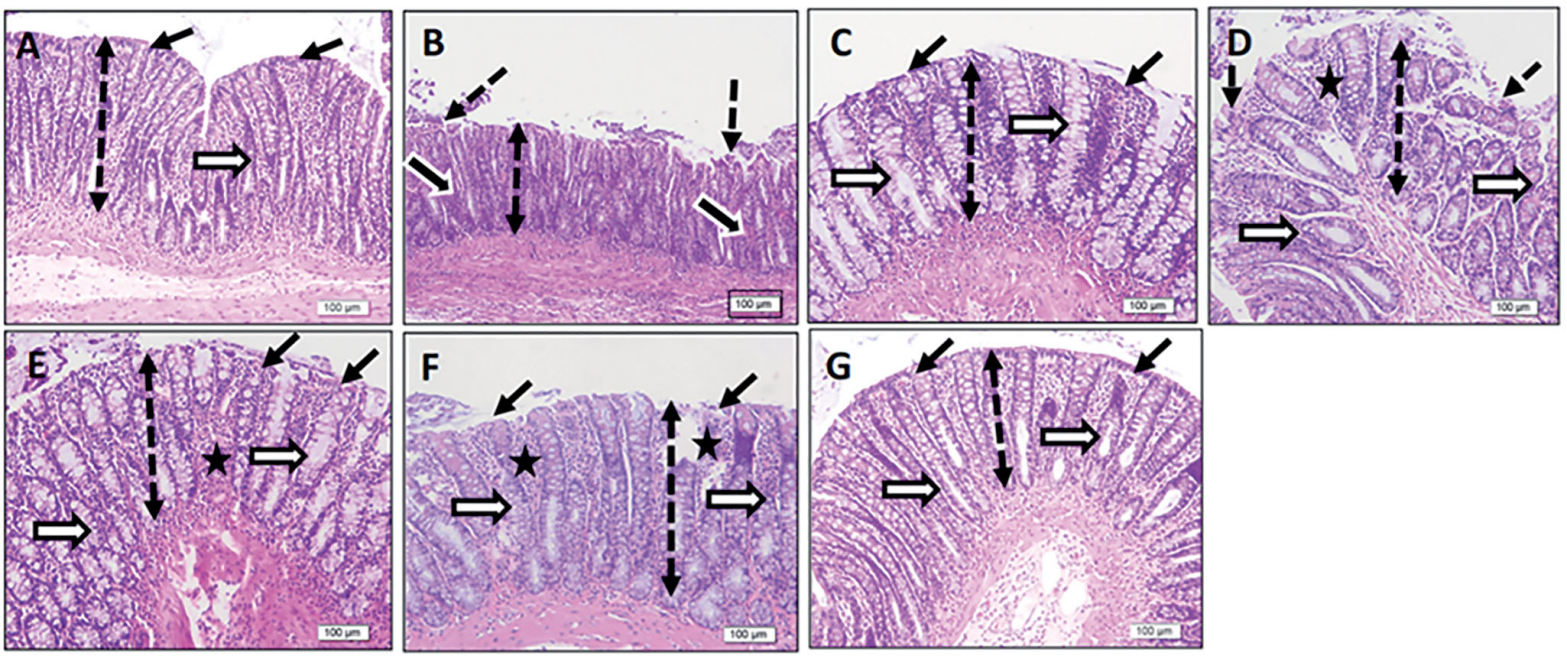
Olmesartan medoxomil self-microemulsifying drug delivery system (OMS) nanoformulation ameliorates histopathological damage in trinitrobenzene sulfonic acid (TNBS)-induced acute colitis in rats. Photomicrographs of colonic sections stained with hematoxylin & Eosin stain (× 20): (A) Normal control group showed intact epithelial surface (thin black arrows), normal-thickness mucosa (double-headed dotted arrow), and normal crypts (white arrows). (B) TNBS-colitic (Positive control) group showed a decreased height of colonic mucosa (double-headed dotted arrow), focal loss of surface epithelium (dotted arrows), dark stained degenerated crypts (thick black arrows), and inflammatory infiltration. (C) Sulfasalazine and (G) Olmesartan medoxomil self-microemulsifying drug delivery system high dose groups showed marked preservation of normal mucosal thickness (double-headed dotted arrow), intact surface epithelium (thin black arrows), normal crypts with increased goblet cells (white arrows), and diminished inflammatory cell invasion. (D) Olmesartan medoxomil low dose group showed mild improvement with focal surface epithelial loss (dotted arrows), a decreased height of colonic mucosa (double-headed dotted arrow), crypt disruption (white arrows), and focal crypt loss (star). (E) Olmesartan medoxomil high dose and (F) Olmesartan medoxomil self-microemulsifying drug delivery system low dose groups showed moderate preservation of normal colonic mucosal structure with a moderately-decreased height of colonic mucosa (double-headed dotted arrow), moderately intact epithelium (thin black arrows), few regions of crypt loss (star), and increased goblet cells (white arrows).

The TNBS-colitic rats showed damaged colonic mucosa with epithelial loss, crypt disruption, inflammatory infiltration, and submucosal edema. All treatments significantly improved these changes. The OML exerted mild improvement, while the OMH and OMSL displayed moderate improvement. The SSZ and OMSH treatments nearly normalized the colonic damage where they showed obvious preservation of normal mucosal thickness, intact epithelium, normal crypts with increased goblet cells, and minimal inflammatory cell infiltration (Figure 4).

### 3.6. Colonic E-cadherin immunostaining

**Figure 5.**
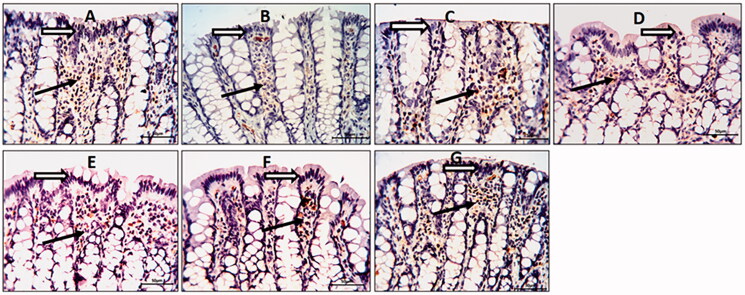
Olmesartan medoxomil self-microemulsifying drug delivery system (OMS) nanoformulation improves the damage of epithelial barrier in trinitrobenzene sulfonic acid (TNBS)-induced acute colitis in rats. Photomicrographs of colonic sections showing immunoexpression of E-cadherin in the surface epithelium (white arrows) and underlying lamina connective tissue cells (black arrows) (× 20): (A) Normal control group showed strong E-cadherin expression, (B) TNBS-colitic (Positive control) group showed reduced E-cadherin expression with damaged surface epithelium. (C) Sulfasalazine and (G) Olmesartan medoxomil self-microemulsifying drug delivery system high dose groups showed marked improvement with preservation of E-cadherin expression. (D) Olmesartan medoxomil low dose group revealed slight preservation of E-cadherin expression. (E) Olmesartan medoxomil high dose and (F) Olmesartan medoxomil self-microemulsifying drug delivery system low dose groups showed moderate preservation of E-cadherin expression.

The TNBS-colitic rats showed a reduced expression level of E-cadherin. All treatments significantly up-regulated the reduced E-cadherin expression. The OML exerted mild improvement, while the OMH and OMSL displayed moderate improvement. The SSZ and OMSH treatments greatly upregulated and nearly normalized the downregulated colonic E-cadherin (Figure 5).

### 3.7. Colonic caspase-3 immunostaining

**Figure 6.**
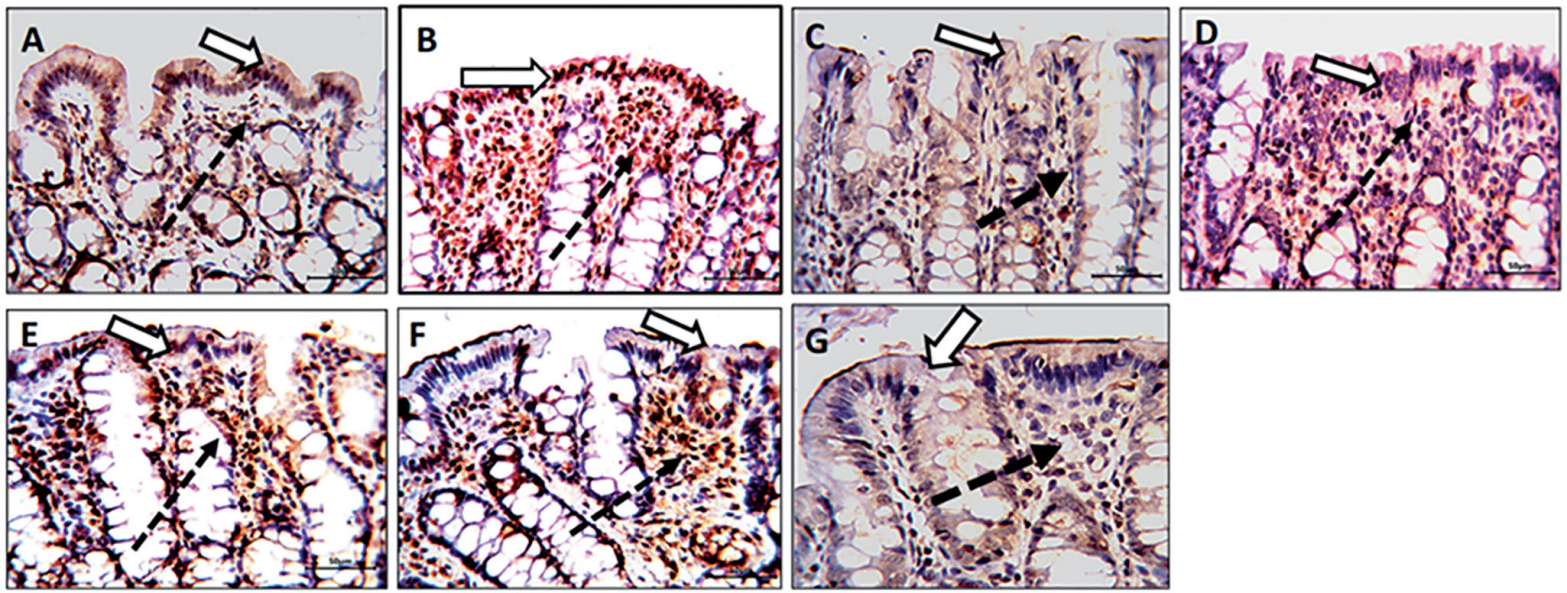
Olmesartan medoxomil self-microemulsifying drug delivery system (OMS) nanoformulation improves apoptosis in trinitrobenzene sulfonic acid (TNBS)-induced acute colitis in rats. Photomicrographs of colonic sections showing immunoexpression of caspase-3 in surface epithelium (white arrows) and underlying lamina connective tissue cells (dotted arrows) (× 20): (A) Normal control group showed a faint reaction. (B) TNBS-colitic (Positive control) group showed a strong reaction with a marked increase of the positively stained cells. (C) Sulfasalazine and (G) Olmesartan medoxomil self-microemulsifying drug delivery system high dose groups showed marked improvement with a marked decrease in the number of the positively stained cells (D) Olmesartan medoxomil low dose group revealed mild improvement. (E) Olmesartan medoxomil high dose and (F) Olmesartan medoxomil self-microemulsifying drug delivery system low dose groups showed moderate improvement.

The TNBS-colitic rats showed promoted immunoexpression of caspase-3. All treatments significantly down-regulated the increased caspase-3 expression. The OML exerted mild improvement, while the OMH and OMSL displayed moderate improvement. The SSZ and OMSH treatments greatly decreased and nearly normalized the upregulated colonic caspase-3 (Figure 6).

### 3.8. Colonic MMP-9 immunostaining

**Figure 7.**
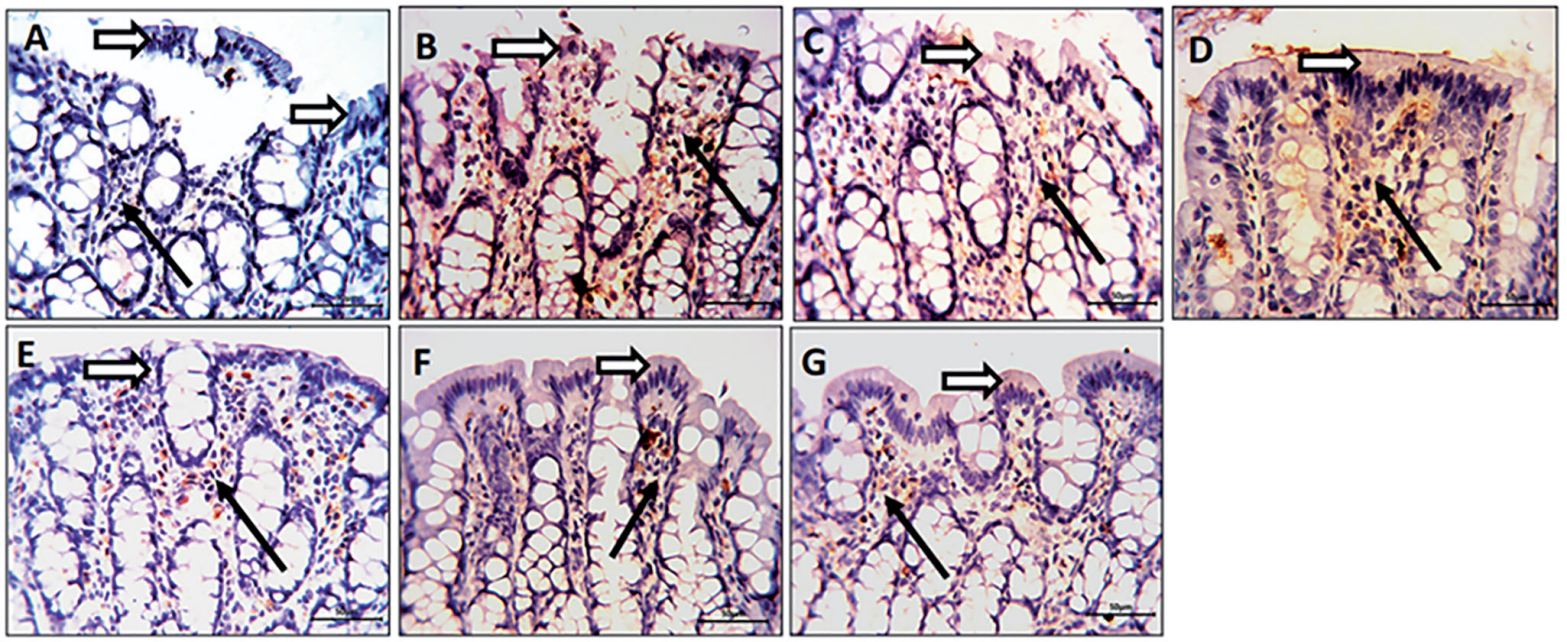
Olmesartan medoxomil self-microemulsifying drug delivery system (OMS) nanoformulation decreases matrix metalloproteinase-9 (MMP-9) overexpression in trinitrobenzene sulfonic acid (TNBS)-induced colitis in rats. Photomicrographs of colonic sections immunostained for MMP-9 in the surface epithelium (white arrow) and connective tissue cells of lamina propria (black arrows) (× 20): (A) Normal control group showed a faint reaction. (B) TNBS-colitic (Positive control) rats showed marked overexpression of MMP-9. (C) Sulfasalazine and (G) Olmesartan medoxomil self-microemulsifying drug delivery system high dose groups showed marked improvement with a marked decrease in the number of positively stained cells. (D) Olmesartan medoxomil low dose revealed mild improvement. (E) Olmesartan medoxomil high dose and (F) Olmesartan medoxomil self-microemulsifying drug delivery system low dose groups showed moderate improvement.

The TNBS-colitic rats displayed a marked overexpression of colonic MMP-9, All treatments significantly down-regulated the MMP-9 overexpression. The OML exerted mild improvement, while the OMH and OMSL displayed moderate improvement. The SSZ and OMSH treatments caused marked improvement with a marked decrease in the number of positively stained cells and nearly normalized the overexpressed colonic MMP-9 (Figure 7).

### 3.9. Protein expression of colonic E-cadherin, Bax, Bcl-2, and cleaved caspase-3

**Figure 8.**
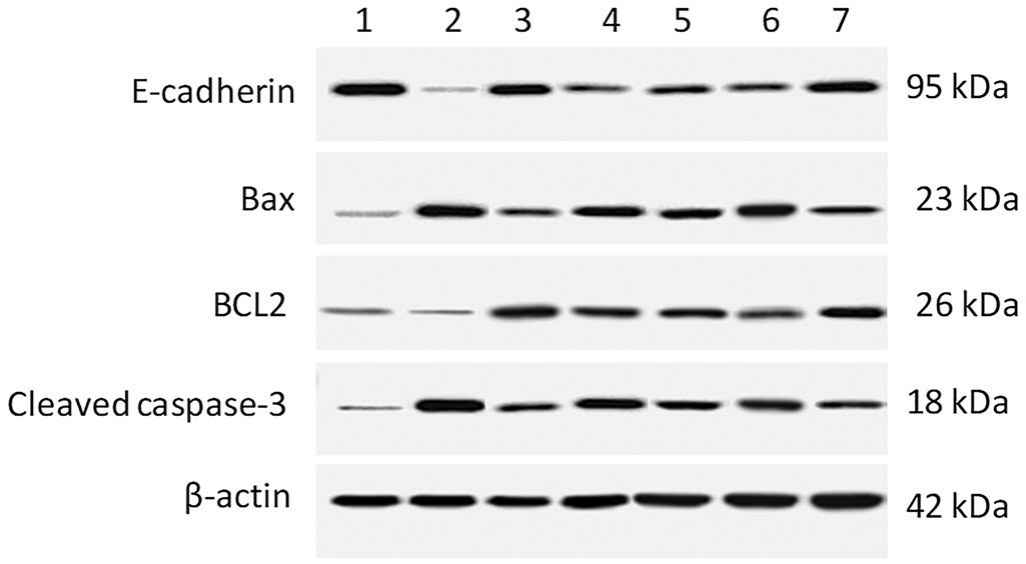
Olmesartan medoxomil self-microemulsifying drug delivery system (OMS) nanoformulation improves apoptosis and endothelial integrity in trinitrobenzene sulfonic acid (TNBS)-induced acute colitis in rats. Protein expressions of E-cadherin, Bcl-2 associated X protein (Bax), and B-cell lymphoma 2 (Bcl-2), and cleaved caspase-3 by Western blot. The figure shows the whole blot after cutting the membrane at molecular weights 80 kDa, 35 kDa, and 20 kDa for E-cadherin (95 kDa), β-actin (42 kDa), Bax (23 kDa), Bcl-2 (26 kDa), and cleaved caspase-3 (18 kDa). (1) Normal control group, (2) TNBS-colitic (Positive control) group, (3) Sulfasalazine (SSZ) group, (4 & 5) Olmesartan medoxomil low & high dose (OML & OMH) groups, (6 & 7) Olmesartan medoxomil self-microemulsifying drug delivery system low & high dose (OMSL & OMSH) groups. The OML exerted mild improvement, the OMH and OMSL displayed moderate improvements, while the SSZ and OMSH exerted the greatest improvements.

The TNBS-induced colitis rats showed a decreased expression level of Bcl-2, increased expression levels of Bax and cleaved caspase-3, and a decreased Bcl-2/Bax ratio. Also, they showed a reduced expression level of E-cadherin. All treatments improved these changes. The OML exerted mild improvement, the OMH and OMSL displayed moderate improvements. The SSZ and OMSH treatments showed marked improvement evidenced by the great increase of expression level of Bcl-2, reduction of expression levels of Bax and cleaved caspase-3, and elevation of the Bcl-2/Bax ratio. Also, they maximally increased the expression level of E-cadherin (Figure 8).

### 3.10. Concentration of olmesartan in plasma and colonic homogenate

**Figure 9.**
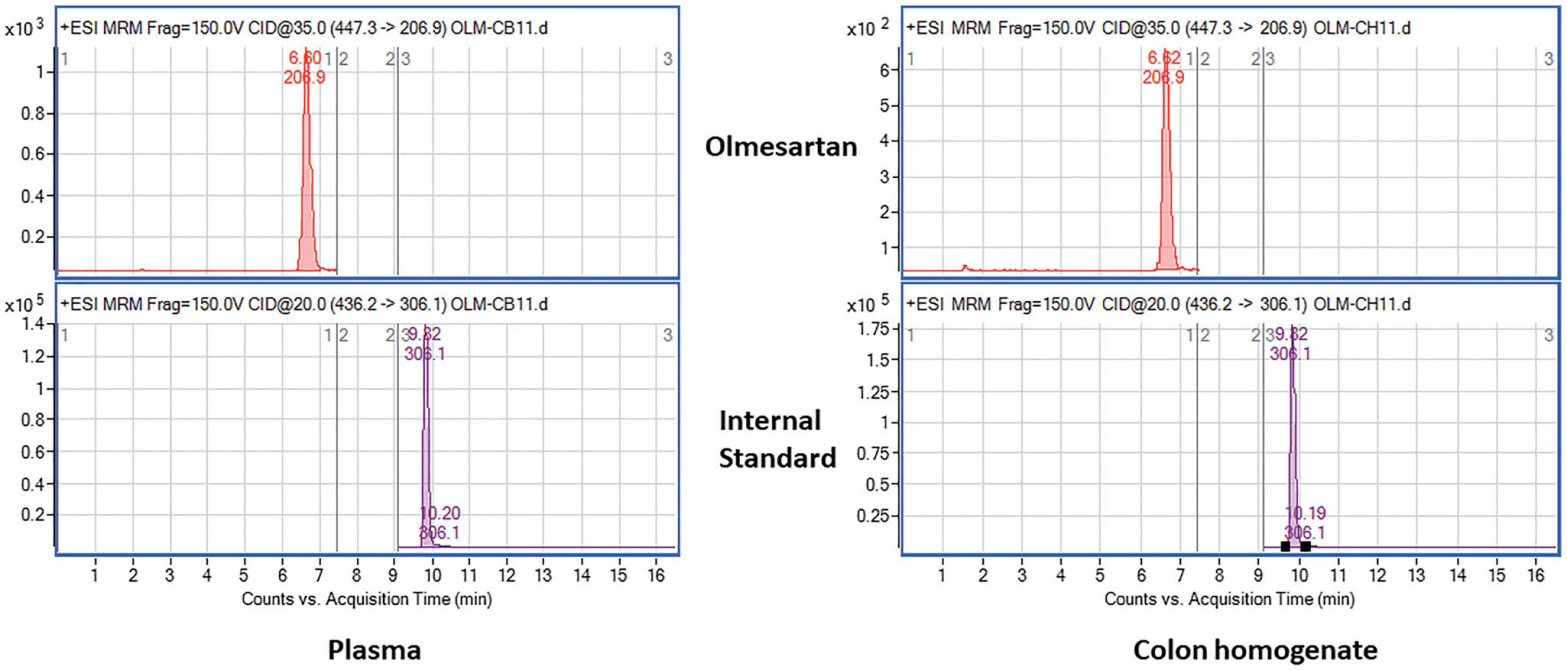
Representative multiple reaction monitoring (MRM) transition chromatograms of olmesartan in the plasma and colonic homogenate.

The OMS achieved higher colonic olmesartan concentrations. Representative MRM transition chromatograms of olmesartan in the plasma and colonic homogenate are shown in Figure 9. Table 3 shows the ratio between colonic free olmesartan concentration and its plasma level (C/P ratio) where the OMSH group showed significant differences from all other groups. The OMH and OMSL groups significantly differed from the OML group with no-significant differences in-between.

**Table 3.** The ratio between colonic free olmesartan concentration and its plasma level (C/P ratio) with olmesartan medoxomil and its nano-formula in trinitrobenzene sulfonic acid-induced acute colitis in rats (*n* = 8).

OML	OMH	OMSL	OMSH
0.91 ± 0.13	4.36 ± 0.33*	5.40 ± 0.31*	8.59 ± 0.36^*, #^

Data are expressed as mean ± SEM. OML: Olmesartan medoxomil low dose, OMH: Olmesartan medoxomil high dose, OMSL: Olmesartan medoxomil self-microemulsifying drug delivery system low dose, OMSH: Olmesartan medoxomil self-microemulsifying drug delivery system high dose. *P < .001 vs. OML, ^#^*P* < .001 vs. OMH, OMSL.

## 4. Discussion

In TNBS-induced colitis, the inflammation initially occurs because ethanol (used to dissolve TNBS) induces damage to intestinal epithelial cells increasing epithelial permeability which allows penetration of microbes and TNBS into the lamina propria. TNBS haptenizes the colonic and gastrointestinal microbial proteins making them immunogenic. This triggers the host immune responses and causes inflammatory infiltration into the broken mucosa (Morampudi et al., 2014). The rectal administration of TNBS significantly increased levels of all pro-inflammatory cytokines including IL-6 and TNF-α with a decrease in the level of the anti-inflammatory cytokine IL-10 (Żyła et al., 2021). In IBD, IL-6 is elevated in both serum and gut tissues (Gross et al., 1992). In TNBS-induced colitis in rats, the colonic mRNA expression and protein level of IL-6 peak at day 7 and then start to gradually decline to normal levels (Wang et al., 2010). The anti-inflammatory, anti-oxidant, and anti-fibrotic beneficial effects of RAS inhibitors in rodent models are mediated through antagonizing effects of Ang II, and upregulating the alternative RAS pathway with the elevation of tissue Ang (1–7) (Ferrario et al., 2005). The human colonic myofibroblast proliferation and collagen secretion were increased by Ang II while decreased by Ang (1–7) (Garg et al., 2020). In the inflamed colonic tissue of patients with IBD, ACEIs or ARBs therapy can inhibit the effects of RAS (Fairbrass et al., 2021). The TNBS-induced elevation of the colonic MPO level indicates neutrophil infiltration (Laroui et al., 2012). Previously it was found that OM decreased neutrophil infiltration as proved by decreasing the colonic MPO activity and improving the histological changes (Biswas, 2016). The increased colonic weight/length ratio in colitis indicates strong intestinal infiltrations and edema (Celinski et al., 2011). The TNBS-colitic rats showed elevated colonic TNF-α levels, epithelial cell necrosis, edema, and neutrophil infiltration (Nakamura et al., 2006). The current results showed that the treatment of rats with OM and its nano formula reversed these changes. The OMSH showed the most significant improvement where it ameliorated DAS by decreasing diarrhea and fecal blood and preserving colonic length. Also, it preserved the colon weight/length ratio, decreased colonic TNF-α, MDA, and MPO, and increased the reduced GSH. Moreover, it improved the macroscopic damage and reversed histopathological and immunohistochemical changes. The OM anti-inflammatory and anti-oxidant effects was previously reported in DSS-induced colitis in rats where OM dose-dependently ameliorated the colonic biochemical and colonic histopathological injuries in a way comparable or even better than that of sulfasalazine (Nagib et al., 2013). Moreover, in acetic acid-induced ulcerative colitis, OM dose-dependently decreased the oxidative stress and inflammatory cytokines. It decreased levels of colonic IL-6, TNF-α neutrophils accumulation, and caspase-3 expression (Saber et al., 2019).

Increased colonic apoptosis has been reported in TNBS colitis (Yue et al., 2001). The oxidant stress triggers the expression of numerous genes responsible for apoptosis (Crespo et al., 2012). In agreement with the current result, OM downregulated the caspase-3 indicating attenuation of colonic apoptosis. The reduction of colonic apoptosis can be attributed to the suppression of oxidative stress because excessive exposure of intestinal mucosa to reactive species under inflammatory conditions increases epithelial apoptosis (Kruidenier et al., 2003). In DSS-induced colitis in female mice, transplantation of male bone marrow-derived mesenchymal stem cells caused regeneration of colon E-cadherin expression indicating restoration of mucosal permeability (Sun et al., 2015). In TNBS-induced Crohn's-like colitis in rats, there was a remarkably down-regulated expression level of E-cadherin and increased apoptosis (Sun et al., 2020). In rats with TNBS-induced colitis, MPO activity got its maximum on days 7 and 10 post-induction and was consistent with the up-regulation of MMP-9 in peripheral and colonic neutrophils which modulates transmural colonic damage (Medina et al., 2006). In rats with Crohn's-like disease, a marked overexpression of colonic MMP-9 mRNA was detected on days 90 and 120 after TNBS administration (Talapka et al., 2016). In acute TNBS-induced colitis in mice, decreasing the up-regulated colonic MMP-9 expression during IBD progression attenuated experimental colonic inflammation (Dutra et al., 2011). Induction of experimental colitis is associated with stress and pain (Salameh et al., 2019; Vecchiarelli et al., 2021), and activation of the renin-angiotensin system (Shi et al., 2016), and a significant reduction in organ blood flow (Konarska et al., 2018). In the gastrointestinal organs, regulation of blood flow is mainly governed by general mechanisms, and thus induction of experimental colitis results in a significant reduction in organ blood flow. Therefore, inhibition of the renin-angiotensin system improves blood flow in gastrointestinal organs protecting them and accelerating the regeneration process (Iwanami et al., 2009; Fändriks, 2011). However, there is no hard clinical evidence supporting this relationship in IBD because although RAS inhibitors have been proven to prevent and ameliorate colitis in animal studies, clinical data in humans are still scarce. Retrospective studies showed that IBD patients using RAS inhibitors had milder disease courses, fewer hospitalizations, and a less corticosteroid use compared with IBD patients not using these medications. However, prospective studies are recommended to assess the effectiveness of these RAS inhibitors in treatment of IBD (Salmenkari et al., 2021).

## 5. Conclusion

The olmesartan medoxomil self-microemulsifying drug delivery system nanoformulation, compared with the same identical doses of the regular olmesartan medoxomil, achieved a higher colonic free olmesartan concentration, and hence more effectively ameliorated the TNBS-induced acute colitis in rats *via* its anti-inflammatory, antioxidant, and anti-apoptotic effects, improving intestinal barrier mechanism, and decreasing mucolytic activity. It more effectively up-regulated the reduced Bcl-2, Bcl-2/Bax ratio, and E-cadherin expression, and down-regulated the overexpressed Bax, cleaved caspase-3, and MMP-9. The olmesartan medoxomil self-microemulsifying drug delivery system low dose exerted effects comparable to olmesartan medoxomil high dose. Sulfasalazine exerted maximal colonic protective effects and almost completely reversed the colonic damage, and olmesartan medoxomil self-microemulsifying drug delivery system high dose showed nearly similar effects with non-significant differences in-between or compared with the normal control group. This might help apply potential safe and relatively inexpensive treatment for IBD. To the best of our knowledge, this is the first report that describes such effects, however, the translation of animal results to clinical reality needs more studies.

## Data Availability

Data are available on reasonable request from the corresponding author.
